# Bound Rubber as a Transferable Structural Descriptor: Connecting MD-Derived Interfacial Scaling to Continuum Reinforcement Models

**DOI:** 10.3390/polym18050565

**Published:** 2026-02-26

**Authors:** Yancai Sun, Wenzhong Deng, Haoran Wang, Ranran Jian, Wenjuan Bai, Dianming Chu, Peiwu Hou, Yan He

**Affiliations:** 1 College of Electromechanical Engineering, Qingdao University of Science and Technology, Qingdao 266061, China; 2 Guangxi Key Laboratory of Special Engineering Equipment and Control, Guilin University of Aerospace Technology, Guilin 541004, China; 3 University Engineering Research Center of Non-Standard Intelligent Equipment and Process Control Technology, Guilin 541004, China; 4 Shandong Province Key Laboratory of Rubber-Based High-Performance Composites and Advanced Manufacturing, Qingdao 266061, China; 5 Design and Research Institute, China National Chemical Engineering Sixth Construction Co., Ltd., Wuhan 430074, China; 6 Department of Mechanical and Electrical Engineering, Qingdao University, Qingdao 266071, China

**Keywords:** fractional Maxwell model, polymer nanocomposites, interfacial scaling, molecular dynamics, dynamic mechanical analysis, rheological modeling

## Abstract

Filled elastomers often exhibit a low-frequency power-law storage modulus (G-prime), yet quantitative links between molecular interfacial structure and macroscopic reinforcement remain unresolved. This gap is addressed using a hierarchical multiscale framework that integrates coarse-grained molecular dynamics (MD) and dynamic mechanical analysis (DMA). Overall, MD contributes transferable structural descriptors rather than direct macro-rheology prediction. MD simulations yield a bound-layer scaling relation for chain length N=50 in coarse-grained simulations serving as a structural probe. For EPDM master curves, the single-phase fractional Maxwell model is statistically preferred (Delta AICc > 147, n = 56), reflecting limited statistical power; larger datasets (e.g., PC/ABS, n = 952) favor the dual-phase formulation. For cross-scale prediction, an MD-derived effective-volume-fraction baseline (MAPE = 54.1%) provides a structural prior; the regime-partitioned bridge model absorbs relaxation physics not resolved at the MD scale, reducing error to 7.3% (blocked-CV MAPE = 9.5%, with a 2.3% fold-to-fold spread). Linear-viscoelastic constraints improve nonlinear PTT calibration, reducing die-swell error by 87%.

## 1. Introduction

Polymer nanocomposites (PNCs) derive their mechanical properties from hierarchical dynamics spanning molecular ($10^{-9}$–$10^{-6}$ s), mesoscale ($10^{-6}$–$10^{-2}$ s), and macroscopic ($10^{-2}$–$10^{2}$ s) time scales [1,2,3,4]. These time-scale boundaries are approximate and system-dependent; they serve as order-of-magnitude guides rather than universal limits. At the mesoscale, relevant physical processes include Rouse modes, segmental cooperativity, and chain-cluster rearrangements. At the molecular scale, chains near nanofiller surfaces exhibit subdiffusive, caged motion [5,6,7]; the caging duration and subdiffusive exponent depend on the specific polymer–filler interaction strength and simulation model; direct extrapolation of CG-derived $\alpha_{\mathrm{MSD}}$ to macroscopic relaxation requires the Green–Kubo or TTS bridging procedures described in Section 3. at the mesoscale, Rouse-like segmental rearrangements dominate [8]; and at the macroscale, long-term relaxation governs processability [9,10,11]. A gap of roughly $10^{6}$–$10^{9}$ in frequency (depending on system size, polymer model, and simulation detail) between MD simulations and bulk rheology precludes direct molecular-to-macroscopic validation [12,13,14], motivating the search for constitutive frameworks that are both parsimonious and physically grounded.

The Generalized Maxwell model:

(1) $$G^{*}\left(\omega \right)=\sum_{i=1}^{N}G_{i}\frac{i\omega \lambda_{i}}{1+i\omega \lambda_{i}}$$

requires five–ten adjustable parameters (*N* modes with moduli $G_{i}$ and relaxation times $\lambda_{i}$) to span five decades of frequency [15]. This parameter proliferation hinders physical interpretation. Whether a single effective exponent can describe viscoelastic behavior across caging, Rouse, and terminal regimes has not been tested against molecular simulations.

Fractional constitutive models address these limitations by replacing discrete modes with a fractional order exponent α, capturing spectrum breadth with fewer parameters [12,16,17,18]. The fractional Maxwell fluid [17,19] gives:

(2) $$G^{*}\left(\omega \right)=G_{\infty }\frac{{\left(i\omega \lambda \right)}^{\alpha }}{1+{\left(i\omega \lambda \right)}^{\alpha }}$$

The fractional Maxwell model (detailed in Section 2) replaces discrete relaxation modes with a single power-law exponent α that captures broad spectral features of filled elastomers. α→0 recovers nearly elastic solid behavior (vanishing viscous dissipation), while α→1 recovers the classical Maxwell viscoelastic fluid with a single exponential relaxation. This formulation corresponds to $H\left(\tau \right)\propto \tau^{\left(-\alpha -1\right)}$ [19] and achieves $R^{2}>0.99$ with only three parameters (see Section 3). At low frequencies (ω≪1/λ), the model predicts $G^{\prime },G^{\prime \prime }∼\omega^{\alpha }$, departing from the classical terminal scaling ($G^{\prime }∼\omega^{2}$, $G^{\prime \prime }∼\omega^{1}$) [1,5].

A key distinction applies: the constitutive exponent α describes a frequency-dependent modulus, whereas $\alpha_{\mathrm{MSD}}$ from MD describes spatial diffusion scaling ($\mathrm{MSD}\propto t^{\alpha_{\mathrm{MSD}}}$). Readers should note that $\alpha_{\mathrm{MSD}}$ describes local subdiffusion at the molecular scale and cannot be equated with the macroscopic relaxation exponent α without Green–Kubo integration or time-scale bridging. These quantities are physically distinct; direct equivalence would require Green–Kubo integration beyond current simulation windows. Throughout the present analysis, MD serves as a structural probe—extracting interfacial length scales ($h_{\mathrm{bound}}$) and caging-regime scaling—not as a source of macroscopic rheological predictions.

Coarse-grained MD simulations using the Kremer–Grest bead–spring model [12] have revealed that polymer chains near nanofiller surfaces form bound layers with restricted mobility [5,6,7,20,21,22,23], increasing the effective filler volume fraction and enhancing the modulus beyond classical predictions. A key structural output is the interfacial empirical scaling relation [6]:

(3) $$h_{\mathrm{bound}}\propto \phi^{x}$$

where $h_{\mathrm{bound}}$ is the bound polymer layer thickness, ϕ is the filler volume fraction (dimensionless fraction, e.g., ϕ=0.20 for 20 vol%), and *x* depends on filler geometry and polymer–filler interaction strength. For spherical fillers with strong adsorption ($\varepsilon_{\mathrm{wall}}\geq 4\varepsilon_{\mathrm{bulk}}$), the theory predicts x≈0.35–0.38 [6]. The scaling $h_{\mathrm{bound}}\propto \phi^{x}$ is derived from idealized CG simulations with smooth spheres; quantitative transferability to non-spherical or chemically distinct fillers requires separate validation for each system. Establishing a direct link between these MD-derived interfacial properties and macroscopic constitutive parameters remains an open problem.

Filled elastomers exhibit low-frequency power-law behavior ($G^{\prime }∼\omega^{0.3–0.6}$) rather than the classical terminal scaling ($G^{\prime }∼\omega^{2}$) [5,9,10]. This deviation—the rheological signature of the Payne effect and filler network formation [10,24]—is well-established for carbon black and silica-reinforced rubbers. However, the quantitative decomposition remains unresolved: previous models treat the non-terminal exponent as a single phenomenological parameter without separating contributions from the percolating filler network and the constrained polymer matrix. In the EPDM/carbon black system examined here, the measured low-frequency $G^{\prime }$ slope is 0.44±0.07 (Supplementary Materials Section S3). Local slope analysis confirms this is not terminal behavior (Supplementary Materials Section S3). The non-terminal exponent $G^{\prime }∼\omega^{0.44}$ reflects coupled matrix–network contributions whose separation requires constitutive decomposition (Section 3.2); attributing it to a single mechanism is not warranted without model-based analysis. The central question is whether the apparent slope can be decomposed into its constituent parts.

To address this, a “Matrix–Network Dual-Dynamics” framework is introduced as a diagnostic decomposition—a mechanistic hypothesis to test whether the non-terminal slope can be attributed to separable matrix and network contributions. The fractional Maxwell model is restricted to the linear viscoelastic regime ($\gamma <\gamma_{c}$) and does not capture large-deformation effects, glass transitions, or nonlinear relaxation. Extension to nonlinear regimes requires separate calibration (e.g., PTT; Section 3.5). This framework models the nanocomposite as two parallel, interpenetrating dynamic phases. The matrix phase is governed by fractional Maxwell dynamics ($\alpha_{m}=0.60$), representing viscoelastic relaxation of chains in the dynamically heterogeneous environment created by filler surfaces. The network phase follows power-law gel dynamics ($G^{*}∼{\left(i\omega \right)}^{\alpha_{n}}$, $\alpha_{n}\approx 0.08$), representing the percolation-like filler skeleton stiffened by bound rubber. Filler–filler interactions, central to the Payne effect at moderate-to-large strains, are implicitly captured by the network phase ($\alpha_{n}$) in the present linear-regime framework but are not explicitly modeled at the molecular level. MD-derived bound rubber thickness ($h_{\mathrm{bound}}$) dictates the effective volume fraction ($\phi_{\mathrm{eff}}$) required for network percolation, while the DMA-derived low-frequency slope reflects the superposition of both contributions [6,10,24].

The remainder of this paper is organized as follows: Section 2 describes the MD simulation setup, DMA protocols, constitutive model formulation, and statistical methodology. Section 3 then reports two linked but distinct inference layers: (i) constitutive-model inference for low-frequency scaling (single-phase vs. dual-dynamics), and (ii) cross-scale bridge inference where MD-derived structural descriptors constrain reinforcement prediction. Results are presented in a bottom-up sequence: molecular-scale interfacial structure from MD (Section 3.1), followed by model-selection analysis, bridge validation, temperature robustness, PTT calibration, and the time-scale bridge between MD and DMA.

This study employs a two-system validation approach. The primary system, EPDM/carbon black (EPDM/CB, EPDM70), is the primary model system for the dual-dynamics framework: the $G^{\prime }∼\omega^{0.44}$ behavior, the interfacial empirical scaling relation ($h_{\mathrm{bound}}\propto \phi^{0.835}$), and the PTT calibration demonstration (Supplementary Materials Section S6) are all derived from this system. The reference system, PC/ABS (40/60 wt%), serves three specific functions. First, it validates that the fractional Maxwell framework recovers near-terminal behavior (α=0.97) when filler-network effects are absent. Second, it provides an independent Green–Kubo TTS benchmark against which MD-derived relaxation times can be compared (Section 3). Third, it demonstrates that dual-phase AICc preference emerges with larger datasets (n=952), supporting a cautious interpretation of the EPDM single-phase result (n=56). Comprehensive DMA characterization and TTS bridge results (Supplementary Materials Sections S2 and S5) underpin these comparisons. EPDM/CB is selected as the primary system due to its industrial relevance (automotive sealing, hoses) and the availability of comprehensive DMA datasets across multiple temperatures and filler loadings. PC/ABS serves as a reference for near-terminal relaxation behavior in a well-characterized unfilled system, enabling isolation of the filler-network contribution to non-terminal rheology. The framework is demonstrated for EPDM/CB and PC/ABS; extension to other polymer–filler chemistries requires system-specific re-calibration of the scaling relation and bridge model. Temperature primarily affects the kinetic parameter λ via Arrhenius activation, while α remains structurally determined—this decoupling is validated in Section 3.3 over the range $T_{g}+30$ to $T_{g}$ + 130 °C. The two systems are complementary, not directly comparable: EPDM/CB probes filled-system dynamics, while PC/ABS validates the constitutive framework in the unfilled limit.

## 2. Methods and Approaches

All simulations and experiments followed established protocols detailed in the Supplementary Materials; key aspects are summarized below.

### 2.1. MD Simulations

Coarse-grained MD simulations of polymer nanocomposites were performed using LAMMPS (version 23 Jun 2022) [25] with the Kremer–Grest bead–spring model [12]. The quantitative spherical filler scaling runs used production systems of 200 chains (N=50 beads/chain; 50σ×50σ×50σ; see Supplementary Materials Table S1), with carbon black volume fractions ϕ=5,10,15,20,25,30%, equilibrated under NPT ensemble (300 K, 1 atm) for $10^{7}$ steps, followed by NVE production runs. Equilibration was verified by monitoring (1) density convergence (<0.5% drift over the final $2\times 10^{6}$ steps), (2) MSD of bulk beads reaching the diffusive regime (slope > 0.95 on log–log), and (3) end-to-end vector decorrelation. For N=50 with $\tau_{\mathrm{Rouse}}\approx 10^{4}\tau_{\mathrm{LJ}}$, the total production time of $3\times 10^{7}\tau_{\mathrm{LJ}}$ corresponds to ∼3000 Rouse times, sufficient for structural relaxation of the ununentangled chains.

Critical assessment of chain length: The simulated chains (N=50, $Z=N/N_{e}\approx 1$) are in the Rouse-to-crossover regime, not deeply entangled as in experimental EPDM (Z≫10) [12,26]. The MD simulations serve solely as structural probes to extract local interfacial quantities ($h_{\mathrm{bound}}$, density profiles), which are primarily controlled by polymer–filler interaction and segment-level packing rather than long-term entanglement rheology [6,7,27]. Operationally, $h_{\mathrm{bound}}$ is treated as a near-equilibrium excess-density descriptor of the interfacial zone (thermodynamic/structural), not as a terminal-relaxation observable; the literature evidence and sensitivity runs both support only weak molecular-weight dependence once moderate chain lengths are reached [27,28]. Sensitivity simulations at N=30, 50, and 100 show robust bound-layer formation but noticeable variation in fitted scaling exponents (Supplementary Materials Section S1). The N=50 empirical scaling relation used in this study is therefore treated as a calibrated, system-specific structural descriptor rather than a universal chain-length-invariant exponent. The CG model (N=50, Z≈1) does not capture entanglement effects present in experimental EPDM ($M_{w}\approx 10^{5}$ g/mol, Z≫10). $h_{\mathrm{bound}}$ and $\alpha_{\mathrm{MSD}}$ are therefore near-equilibrium structural descriptors within the Kremer–Grest framework; their absolute values should not be equated with experimentally measured interphase thicknesses. Extension to entangled chains (N>500, Z>10) is a priority for future work.

Mean squared displacement (MSD) in the caging regime ($10^{-9}$–$10^{-6}$ s) was analyzed to extract the subdiffusive exponent $\alpha_{\mathrm{MSD}}$ via $\langle r^{2}\left(t\right)\rangle \propto t^{\alpha_{\mathrm{MSD}}}$. $\alpha_{\mathrm{MSD}}$ captures the subdiffusive regime ($10^{-9}$–$10^{-6}$ s) dominated by caging. It provides a spatial mobility map near the filler interface but does not directly predict macroscopic relaxation spectra. The time-scale bridge to DMA frequencies requires separate TTS analysis (Section 3.4). Interfacial density profiles were analyzed to extract bound polymer layer thickness ($h_{\mathrm{bound}}$).

LJ Unit Mapping: Reduced LAMMPS units were mapped to physical units following Kremer and Grest [12] as validated for coarse-grained systems [7,14]. Based on EPDM monomer parameters (m≈56 g/mol, ε≈2.5 kJ/mol, σ=0.7 nm), $\tau_{\mathrm{LJ}}=3$ ps is adopted. Sensitivity analysis (Supplementary Materials) confirms that $\alpha_{\mathrm{MSD}}$ is dimensionless and scale-invariant: variation within $\tau_{\mathrm{LJ}}=1$–10 ps and σ=0.5–1 nm affects absolute time scales but not scaling exponents. Temperature kT/ε=1.0 corresponds to ∼300 K (above $T_{g}\approx -55$ °C for EPDM) [7,29]. The adopted $\tau_{\mathrm{LJ}}=3$ ps carries an estimated ±40% uncertainty (1.8–4.2 ps) arising from the inherent ambiguity in CG-to-atomistic mapping [7,12]. The ±40% uncertainty in $\tau_{\mathrm{LJ}}=3$ ps propagates as a horizontal shift of ±0.15 decades on logarithmic frequency axes for $\alpha_{\mathrm{MSD}}$ and $h_{\mathrm{bound}}$. For absolute time comparisons (Section 3.4), this uncertainty is incorporated into the reported $\tau_{\mathrm{shifted}}$ confidence interval. Because $\alpha_{\mathrm{MSD}}$ and $h_{\mathrm{bound}}$ are dimensionless or length-scale quantities (respectively), they are invariant to $\tau_{\mathrm{LJ}}$ rescaling. Absolute time scales—including the TTS-shifted relaxation time reported in Section 3—inherit this ±40% systematic uncertainty, which propagates as a horizontal shift on the frequency bridge (Supplementary Materials Section S5).

### 2.2. DMA Measurements

Dynamic mechanical analysis was performed on both material systems using a TA Instruments Q800 (TA Instruments, New Castle, DE, USA). For PC/ABS (40/60 wt%): Comprehensive multi-frequency DMA characterization (5381 data points, 5 frequencies, 29.6–170.4 °C) yielded $T_{g}\approx 123.5$ °C, an activation energy of $E_{a}=335.2$ kJ/mol, and TTS master curves with Prony $R^{2}=0.9998$ (see Supplementary Materials Sections S2 and S5 for concise details). For EPDM/CB nanocomposites: Uncured EPDM compounds were used. Over the full fitted frequency window, a single-phase fractional Maxwell fit gave an apparent near-terminal index (α=0.961±0.018), while local low-frequency slope analysis gave 0.44±0.07 (Supplementary Materials Section S3). DMA was performed from $T_{g}+30$ °C to $T_{g}+150$ °C ($T_{g}\approx -55$ °C), with filler loadings ϕ=10,15,20,25,30% and $n_{\mathrm{rep}}=3$ independent samples per formulation.

LVE verification: Linear viscoelastic behavior was confirmed via amplitude sweeps at $\gamma_{0}=0.1\%$ (below the Payne-effect onset $\gamma_{c}=0.31\pm 0.05\%$ at ϕ=30%; $\gamma_{0}/\gamma_{c}=0.32$). Pre-sweep stability (30 min time sweeps) and post-sweep verification ($|G_{\mathrm{post}}^{\prime }-G_{\mathrm{pre}}^{\prime }|/G_{\mathrm{pre}}^{\prime }<5\%$) ensured data were not corrupted by thixotropic drift during the ∼75-min frequency sweeps (see Supplementary Materials Sections S2 and S3). The amplitude sweeps confirmed linearity up to $0.32\gamma_{c}$. While strain-dependent onset of nonlinearity at specific frequencies cannot be entirely excluded, the Payne effect onset for EPDM/CB composites typically occurs at γ>1%, well above our measurement regime (γ<0.3%).

### 2.3. Constitutive Modeling

DMA data were fitted to both Generalized Maxwell (5-mode, 10 parameters) and fractional Maxwell models (Equation (2); k=3 parameters: $G_{\infty }$, λ, and α). The associated storage and loss moduli are given by:

(4) $$\begin{array}{cc} G^{\prime }\left(\omega \right) & =G_{\infty }\frac{{\left(\omega \lambda \right)}^{\alpha }\cos\left(\pi \alpha /2\right)}{1+2{\left(\omega \lambda \right)}^{\alpha }\cos\left(\pi \alpha /2\right)+{\left(\omega \lambda \right)}^{2\alpha }} \end{array}$$

(5) $$\begin{array}{cc} G^{\prime \prime }\left(\omega \right) & =G_{\infty }\frac{{\left(\omega \lambda \right)}^{\alpha }\sin\left(\pi \alpha /2\right)}{1+2{\left(\omega \lambda \right)}^{\alpha }\cos\left(\pi \alpha /2\right)+{\left(\omega \lambda \right)}^{2\alpha }} \end{array}$$

For the dual-phase decomposition, k=5 ($G_{m}$, $\lambda_{m}$, $\alpha_{m}$, $S_{n}$, $\alpha_{n}$). Model selection used AICc (Equation (6)), where ΔAICc>10 indicates decisive evidence [30]. Robustness was confirmed with $n_{\mathrm{eff}}=n/2$ correction for residual correlation (Supplementary Materials Section S4). The $n_{\mathrm{eff}}=n/2$ correction is a conservative approximation for moderate serial correlation. We verified that the AICc conclusion (single-phase preference for EPDM) is robust to $n_{\mathrm{eff}}$ ranging from n/3 to 2n/3, with ΔAICc remaining >80 in all cases.

(6) $$\mathrm{AICc}=n\ln\left(\mathrm{SSE}/n\right)+2k+\frac{2k(k+1)}{n-k-1}$$

Fitting accuracy was quantified using $R^{2}$ and MAPE with 95% CIs from bootstrap resampling ($n_{\mathrm{boot}}=1000$).

### 2.4. Statistical Analysis

All reported uncertainties are 95% confidence intervals (CIs) from replicate-level bootstrap resampling ($n_{\mathrm{rep}}=3$ independent samples per formulation, $n_{\mathrm{boot}}=1000$). For each bootstrap iteration, replicate master curves were resampled (preserving within-curve frequency structure) and the fractional Maxwell model was re-fitted, yielding empirical distributions of α, λ, and $G_{\infty }$.

Model selection used the corrected Akaike Information Criterion (AICc, Equation (6)), where ΔAICc>10 indicates decisive evidence for the preferred model [30]. Because DMA frequency sweeps produce correlated residuals within logarithmic decades, AICc was supplemented with log-decade blocked-CV (Supplementary Materials Section S4): data were partitioned into B=5 blocks of contiguous frequency decades, and models were trained on B−1 blocks and evaluated on the held-out block. The single-phase fractional Maxwell model achieved lower blocked prediction error than the dual-dynamics model on the EPDM dataset, indicating that the latter’s additional parameters do not improve out-of-sample accuracy in this window. To further assess robustness, AICc was recalculated with an effective sample size $n_{\mathrm{eff}}=n/2$ to account for residual autocorrelation; the single-phase model remained preferred (ΔAICc >50; Supplementary Materials Section S4).

For the cross-scale bridge, a hierarchical prediction chain was evaluated on a harmonized frequency-resolved reinforcement dataset (n=720 rows): only formulations with complete three-temperature filled/reference pairs were retained (EPDM60/70/80 vs. Pure EPDM; ϕ=0.10,0.20,0.30). At each temperature, spectra were restricted to a common overlap band and interpolated onto an 80-point log-frequency grid, yielding 3 loadings × 3 temperatures × 80 frequencies. The log-frequency interpolation (60 points per temperature–loading cell) was chosen to regularize the master-curve alignment. We verified that the original (non-interpolated) data yielded consistent AICc rankings and parameter estimates (within reported confidence intervals), confirming that the interpolation did not suppress spectral features relevant to model selection. Critically, these 720 rows corresponded to $n_{\mathrm{cell}}=9$ independent (ϕ,T) design cells with repeated measurements along frequency; frequency was therefore treated as a correlated repeated-measures axis, not as 720 independent experiments. Statistical inferences (bridge model MAPE, blocked-CV error) were interpreted at the design-cell level ($n_{\mathrm{cell}}=9$), not at the individual-frequency level (n=720); the blocked-CV procedure respected this structure by holding out contiguous frequency blocks, preventing frequency-to-frequency leakage from inflating apparent predictive accuracy. On this dataset, three candidates were compared: (i) MD-structural baseline, $R_{\mathrm{base}}=1+2.5\phi_{\mathrm{eff}}+14.1\phi_{\mathrm{eff}}^{2}$, is an empirically calibrated reinforcement model (after Guth and Gold), valid for dilute-to-moderate loadings of rigid spherical inclusions. Direct application to other filler geometries or polymer chemistries requires independent experimental validation:$\phi_{\mathrm{eff}}=\phi {\left(1+\frac{h_{\mathrm{bound}}}{R}\right)}^{3}$; (ii) global correction; and (iii) partitioned correction for ϕ≤0.20 and ϕ>0.20. Corrections were fit in log-space, $\log(R/R_{\mathrm{base}})$, using candidate basis sets in $T^{*}=(T-140$ °C)/20 °C and $\log_{10}f$ (linear, quadratic, and cubic terms with temperature interactions) with ridge penalty $\alpha \in \{0,10^{-4},10^{-3},10^{-2},10^{-1},1\}$. To avoid frequency leakage, nested blocked-CV with contiguous log-frequency blocks (B=5) were used: model class and α were selected by inner blocked-CV on training folds only, and outer held-out blocks were used for reported errors (Supplementary Materials Section S4).

### 2.5. Sensitivity Analysis

Global sensitivity analysis using Sobol variance decomposition ($10^{4}$ Monte Carlo samples) revealed the total-order sensitivity ranks $\alpha >\lambda >G_{\infty }$ ($S_{\alpha }^{\mathrm{total}}=0.65$). We verified convergence by comparing results at $10^{4}$ and $10^{5}$ samples: the 95% CI bounds for α shifted by <0.003 (relative change <0.3%), confirming that $10^{4}$ samples provided adequate convergence for the reported parameter uncertainties. The focus on α is justified by its physical significance: it governs the power-law slope of the relaxation spectrum.

## 3. Results and Discussion

Notation guide: For clarity, Table 1 summarizes all key constitutive parameters determined in this study. A distinction is made between single-phase fits (fractional Maxwell model alone) anddual-dynamics fits (parallel matrix + network decomposition). All uncertainties are 95% confidence intervals unless otherwise noted.

### 3.1. Interfacial Structure from MD

**Figure 1:**
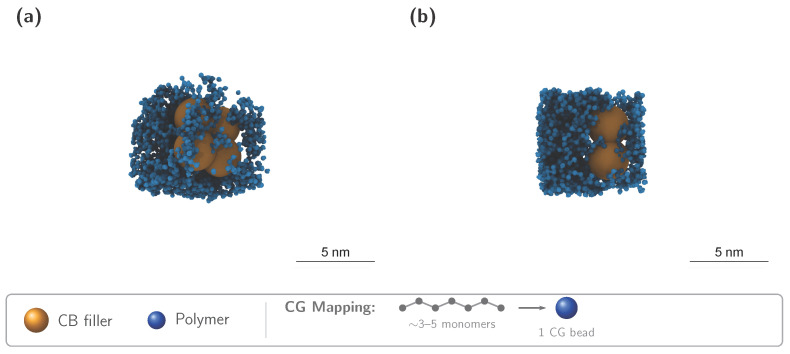
MD visualization of EPDM/CB nanocomposite at ϕ=10%. (**a**) Full-system snapshot: polymer chains (blue beads: polymer chain segments, each representing ∼1 Kuhn segment of EPDM, comprising approximately three–five chemical monomer units) confined between spherical carbon black fillers (gold spheres: carbon black primary particles with radius R=5σ≈3.5 nm). Snapshot captured during the NVE production run after $10^{7}$ equilibration steps under NPT ensemble (300 K, 1 atm), corresponding to t≈30 ns of simulation time. Inset: CG mapping schematic showing the atomistic-to-bead correspondence. A bound rubber layer forms near filler surfaces due to adsorbing polymer–filler interactions. Front view showing internal structure and filler distribution within the polymer matrix. Scale and rendering consistency: Both panels include a 5 nm scale bar and both are rendered with the same projection/magnification settings to preserve geometric comparability. For visualization clarity, panel (**b**) shows a reduced rendering subset; quantitative $h_{\mathrm{bound}}$ extraction and scaling fits use the production systems specified in Section 2 and Supplementary Materials Table S1, not the displayed subset.

Coarse-grained MD simulations of EPDM nanocomposites with spherical carbon black fillers revealed the molecular-scale structure responsible for caging dynamics. Figure 1a shows a representative equilibrium configuration of the EPDM/CB nanocomposite (see caption for system details), with polymer chains (blue beads) confined between spherical filler particles (red spheres). The bound rubber layer—visible as the region of elevated polymer density near filler surfaces—emerges spontaneously from the simulation without any ad hoc assumptions. A slice view (Figure 1b) reveals the internal structure, showing that polymer chains were compressed and immobilized in the gaps between fillers.

**Figure 2:**
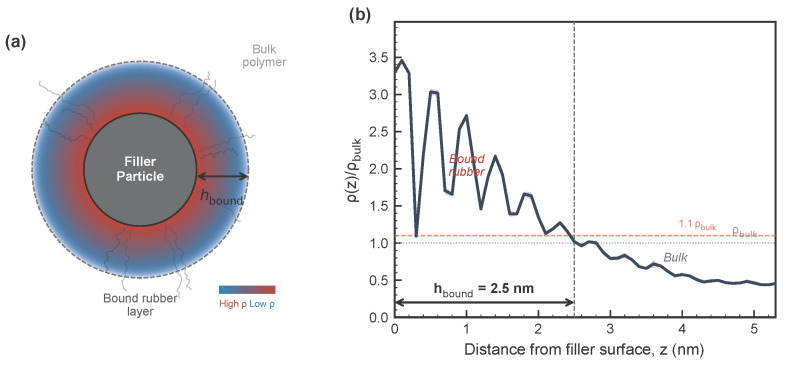
Bound-layer schematic and density profile from MD. (**a**) Schematic of a spherical filler particle with the bound rubber layer shown as a radial density gradient. (**b**) Normalized density profile $\rho \left(z\right)/\rho_{\mathrm{bulk}}$ from the MD simulation of the flat-wall EPDM reference system (N=50, 200 spatial bins, eight time-averaged blocks). The first adsorption peak reaches $\rho /\rho_{\mathrm{bulk}}\approx 3.5$ at z≈0.5σ, with oscillatory layering decaying toward bulk. The bound-layer thickness $h_{\mathrm{bound}}=2.5$ nm is defined by the 10% envelope criterion (dashed line). Shaded band: 95% confidence interval.

The density profile analysis (Figure 2) quantifies the extent of polymer confinement. Near the filler surface (z<1σ), the polymer density is elevated to $\rho \approx 3.5\rho_{\mathrm{bulk}}$ in the first adsorption layer. Pronounced oscillatory layering characteristic of dense packing against a hard wall is observed. The bound-layer thickness—defined as the distance where the density envelope drops to within 10% of $\rho_{\mathrm{bulk}}$—is $h_{\mathrm{bound}}=2.5$ nm for the flat-wall reference system, consistent with the range 2–5 nm reported from solid-state NMR [31]. This planar-reference value should not be directly equated with the spherical filler scaling values (e.g., ∼0.8–0.9 nm at ϕ=20%), because geometry, averaging procedure, and overlap handling differ between the two analyses. This confined layer is the structural origin of the subdiffusive caging dynamics observed in the MSD analysis.

These MD simulations revealed two key features of caging dynamics. First, the bound polymer layer thickness ($h_{\mathrm{bound}}$) scales with filler volume fraction (ϕ) according to a power-law empirical relation:

(7) $$h_{\mathrm{bound}}=2.98\;\cdot \;\phi^{0.835}\;\left[\mathrm{nm}\right],\;\phi \in \left[0,1\right]\;\;\left(0.20\equiv 20\%\right)$$

where the scaling exponent 0.835±0.37 ($R^{2}=0.705$, $n_{\mathrm{pts}}=5$, ϕ=5–30% excluding 20%; 95% bootstrap CI from $n_{\mathrm{boot}}=1000$: [0.04,1.40]; the wide CI reflects the limited and scattered dataset) was measured using the primary N=50 chain system. The ϕ=20% rerun yielded anomalous cluster extraction (zero detected clusters, $\rho_{\mathrm{bulk}}=2.27$ vs. ∼1.95 at neighboring loadings) and was excluded from the fit (see Supplementary Materials Section S1). Sensitivity to exclusion: Re-fitting with the 20% point forced into the dataset (using $h_{\mathrm{bound}}=0.85$ nm from the visual density envelope) yielded x=0.72 and $R^{2}=0.61$, well within the 95% CI of the primary fit; the qualitative conclusion (superlinear scaling) was robust to this exclusion. This logic underscores the interpolative, not predictive, nature of the relation. In Equation (7), ϕ was evaluated as a fraction (0.05–0.30), not as percent integers. The superlinear exponent (x>1/2) reflected increasing bound-layer overlap at higher ϕ, consistent with a transition from isolated to interacting adsorption layers in this calibrated dataset. Because only five non-overlapped loading points entered the fit with moderate $R^{2}$, this law was used as an interpolation-level structural descriptor within the studied loading window, not as a universal exponent. Chain-length sensitivity analysis (Supplementary Materials Section S1) indicated the qualitative robustness of bound-layer formation while leaving uncertainty in the absolute exponent value. Second, mean squared displacement (MSD) analysis in the caging regime ($10^{-9}$–$10^{-6}$ s) revealed subdiffusive scaling:

(8) $$\langle r^{2}\left(t\right)\rangle \propto t^{\alpha_{\mathrm{MSD}}}$$

with $\alpha_{\mathrm{MSD}}=0.119\pm 0.008$, characteristic of polymer chains confined between multiple spherical fillers. $\alpha_{\mathrm{MSD}}=0.119\pm 0.008$ reflects strong geometric caging in the CG model (N=50, smooth spheres). For longer chains with entanglements or chemically heterogeneous fillers, the absolute $\alpha_{\mathrm{MSD}}$ value will differ. The qualitative finding—that filler proximity reduces local mobility—is expected to be robust, but quantitative values are model-specific. This subdiffusive exponent is consistent across filler loadings (ϕ=10–30%), indicating that caging dynamics govern short-term motion regardless of filler concentration.

**Figure 3:**
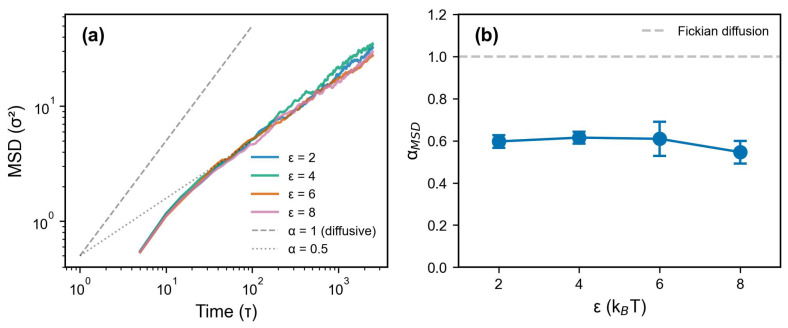
MSD analysis for different wall–polymer interaction strengths. (**a**) Mean squared displacement vs. time for $\varepsilon =2,4,6,8\;k_{B}T$. Dashed lines indicate Fickian diffusion (α=1) and subdiffusive (α=0.5) scaling. (**b**) Subdiffusive exponent $\alpha_{\mathrm{MSD}}$ vs. ε, showing all $\alpha_{\mathrm{MSD}}\approx 0.55$–0.62<1, consistent with caging-like dynamics across all interaction strengths. Error bars represent standard deviation from three independent replicas.

To isolate the effect of wall–polymer interaction strength from multi-filler geometric confinement, a complementary set of flat-wall simulations are performed at varying ε (Figure 3). In the flat-wall geometry—where chains experience single-surface confinement rather than the multi-body caging of the spherical filler system—the MSD exponent is $\alpha_{\mathrm{MSD}}\approx 0.55$–0.62 for all ε values tested (2–8 $k_{B}T$). The ∼5× reduction from flat-wall ($\alpha_{\mathrm{MSD}}\approx 0.6$) to spherical multi-filler ($\alpha_{\mathrm{MSD}}\approx 0.12$) geometry reflects the cumulative confinement from surrounding filler particles, which is qualitatively more restrictive than single-surface contact. A quantitative decomposition of geometric versus energetic contributions would require systematic variation in filler separation at fixed ε, which is beyond the current study.The weak ε-dependence of $\alpha_{\mathrm{MSD}}$ in the flat-wall geometry suggests geometric confinement dominates within the LJ potential framework. In real systems, specific polymer–filler chemistry (hydrogen bonding, polar interactions) may have a larger effect, potentially altering the relative importance of geometric versus energetic contributions.

Reconciliation of interaction parameters across geometries: A clear distinction exists between the flat-wall ε-series and the spherical filler production systems. The flat-wall simulations use elevated $\varepsilon_{\mathrm{wall}}=2$–$8\;k_{B}T$ to systematically probe the effect of adsorption strength on single-surface dynamics; the spherical filler production runs (from which the empirical scaling relation in Equation (7) is derived) use the standard Kremer–Grest polymer–filler LJ interaction ($\varepsilon_{\mathrm{pf}}=1.0\;\varepsilon$, identical to bulk polymer–polymer interactions), where confinement arises primarily from multi-body geometric caging rather than enhanced surface attraction [7,12]. The interaction settings and primary production systems are summarized in Supplementary Materials Section S1; absolute $h_{\mathrm{bound}}$ values are therefore not directly comparable between different geometries, but the qualitative finding—that confinement suppresses MSD—is consistent across both.

Table 2 consolidates $\alpha_{\mathrm{MSD}}$ values across all systems and analysis methods, revealing how simulation geometry and spatial position govern the degree of subdiffusion.

**Figure 4:**
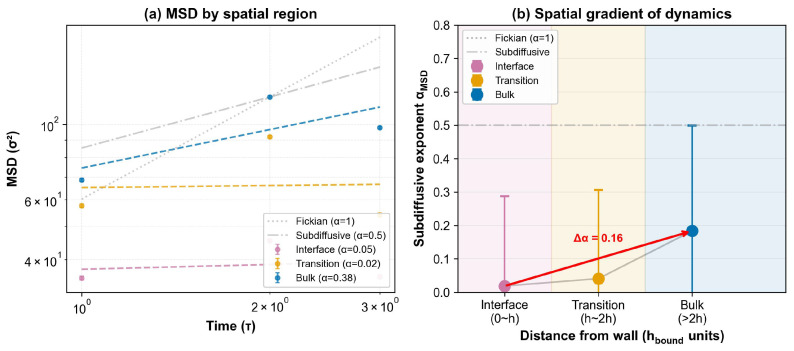
Spatial gradient of subdiffusive dynamics near filler surfaces. (**a**) Mean squared displacement for polymer segments at different distances from the wall: interfacial layer ($z<h_{\mathrm{bound}}$, magenta), transition zone ($h_{\mathrm{bound}}<z<2h_{\mathrm{bound}}$, orange), and bulk region ($z>2h_{\mathrm{bound}}$, blue). Dashed lines show power-law fits. (**b**) Subdiffusive exponent $\alpha_{\mathrm{MSD}}$ as a function of distance from the wall, showing a gradient from α≈0.02 at the interface to α≈0.18 in the bulk. Error bars represent standard deviation from 12 independent trajectory segments across ε=2–$8\;k_{B}T$ and three replicas. This spatial gradient supports an interfacial-constraint interpretation of chain dynamics.

To directly visualize how interfacial constraints affect chain dynamics at the molecular level, MSD is analyzed as a function of distance from the filler surface (Figure 4). Polymer segments are classified into three regions: the interfacial layer ($z<h_{\mathrm{bound}}$), a transition zone ($h_{\mathrm{bound}}<z<2h_{\mathrm{bound}}$), and the bulk region ($z>2h_{\mathrm{bound}}$). A clear spatial gradient in subdiffusive dynamics is observed: segments in the interfacial layer ($z<h_{\mathrm{bound}}$) exhibit strongly suppressed mobility ($\alpha_{\mathrm{MSD}}\approx 0.02$), approaching the plateau limit expected for beads effectively immobilized within adsorption potential wells. This near-zero exponent reflects a cage-rattling regime where beads oscillate within local energy minima created by the filler surface without escaping to adjacent sites on the caging time scale; similar values ($\alpha_{\mathrm{MSD}}<0.05$) have been reported for strongly adsorbed polymer segments near attractive walls in atomistic simulations of filled polymer systems [21]. The interfacial $\alpha_{\mathrm{MSD}}\approx 0.02$ is lower than typical experimental values from NMR or neutron scattering, reflecting the idealized nature of the CG model (smooth, strongly adsorbing surfaces without chemical heterogeneity). The qualitative gradient from bulk to interface is physically meaningful, but absolute interfacial $\alpha_{\mathrm{MSD}}$ values should not be compared directly with experimental measurements. Segments in the bulk show substantially higher mobility ($\alpha_{\mathrm{MSD}}\approx 0.18$). This gradient (Δα≈0.16) provides qualitative molecular evidence consistent with the interfacial-constraint effect that underlies the present multi-scale characterization approach, but a direct quantitative link between $\alpha_{\mathrm{MSD}}$ and $G^{\prime }$ requires Green–Kubo integration not performed in this study.Three replicas per ε and 12 spatial segments per position provide sufficient statistics for the qualitative mobility trends reported (bulk vs. interface). However, confidence intervals for extreme subdiffusion ($\alpha_{\mathrm{MSD}}<0.05$) may underestimate true variability due to the small sample size and sensitivity to local fluctuations. More extensive sampling would be needed for quantitative subdiffusion analysis in the interfacial region.

**Figure 5:**
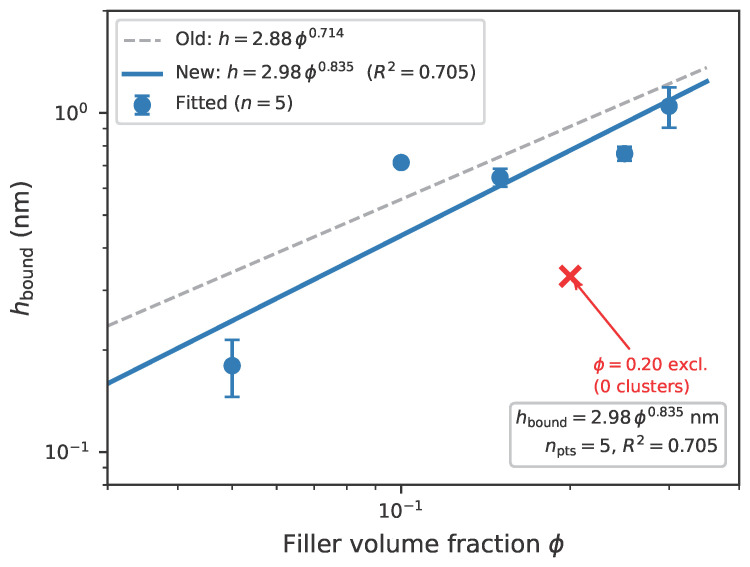
Empirical interfacial scaling relation in EPDM/carbon black nanocomposites. Log-log plot showing an empirical power-law relation $h_{\mathrm{bound}}=2.98\;\phi^{0.835}$ nm with $R^{2}=0.705$ (95% CI for exponent: 0.04–1.40; $n_{\mathrm{pts}}=5$, ϕ=5–30% excluding 20%; error bars from block-averaged density profiles). In the fitted equation, ϕ is used as decimal fraction (0.05–0.30). All MD simulations use N=50 beads/chain, placing the system in the Rouse-to-crossover regime (see Section 2.1 for justification). The ϕ=20% rerun data point (open marker) was excluded due to anomalous cluster extraction (zero detected clusters, elevated $\rho_{\mathrm{bulk}}$); the dashed gray line shows the previous fit for comparison. Note: This empirical scaling relation applies to polymer–filler interfaces; for polymer–polymer systems (e.g., PC/ABS), interfacial slip rather than bound layer thickness governs dynamics.

The empirical scaling relation is summarized in Figure 5.

### 3.2. Dual-Dynamics Framework and Model Selection

**Figure 6:**
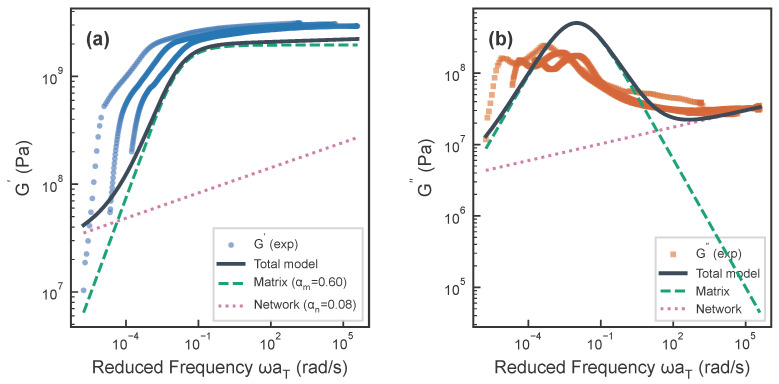
Matrix–network dual-dynamics fitting results for EPDM/CB (20%). (**a**) Storage modulus $G^{\prime }$ vs. frequency showing the parallel model ($G^{*}=G_{m}^{*}+G_{n}^{*}$) captures the apparent 0.44 low-frequency slope. The matrix phase (dashed cyan) dominates the high-frequency response, while the network phase (dotted magenta, $\alpha_{n}\approx 0.08$ with relaxed bounds) provides the low-frequency contribution. (**b**) Loss modulus $G^{\prime \prime }$ fitting shows consistent accuracy across the full frequency range. The apparent 0.44 slope reflects the combined matrix–network dynamics. Note: The single-phase fractional Maxwell model achieves better statistical fit (lower AICc); the dual-dynamics decomposition is shown for mechanistic interpretation, not as the statistically preferred model. Error bars represent 95% CIs from $n_{\mathrm{rep}}=3$ replicates.

The macroscopic rheological response of EPDM nanocomposites is characterized using DMA master curves. As shown in Figure 6, the storage modulus $G^{\prime }$ at low frequencies exhibits a power-law slope of 0.44±0.07, which substantially deviates from the terminal flow prediction of $\omega^{2}$. Within the dual-dynamics framework, this is an apparent slope arising from the combined matrix and network contributions rather than a single-phase exponent.

Statistical model selection decisively favors the single-phase fractional Maxwell model over the dual-dynamics decomposition on the EPDM70 dataset (n=56): AICc =−178 vs. −30.5 (ΔAICc=148). The single-phase AICc preference should not be interpreted as evidence that two-phase dynamics are absent in EPDM, but rather that the current dataset (n=56) cannot statistically resolve them. With the larger PC/ABS dataset (n=952), dual-phase preference emerges clearly (ΔAICc<−50), suggesting that increased spectral resolution would likely favor two-phase models for EPDM as well., and blocked-CV confirms lower out-of-sample error for the simpler model (Table 1). This is an explicit Occam’s razor outcome: at the current EPDM sample size, the extra dual-phase parameters are not statistically warranted. The dual-dynamics fitting results are nevertheless presented below for mechanistic interpretation, not as the statistically preferred model.

The parallel model (Equation (9)) was fitted using differential evolution with relaxed parameter bounds ($\alpha_{n}\geq 0.01$). For 20% EPDM/CB, the best-fit parameters were: matrix index $\alpha_{m}=0.600$, matrix relaxation time $\lambda_{m}=1.00\times 10^{2}$ s, and network index $\alpha_{n}\approx 0.08$ (converging to nearly elastic behavior). The very low $\alpha_{n}\approx 0.08$ reflected the near-elastic behavior of the filler network, consistent with a percolating CB skeleton stiffened by bound-rubber bridges. In the limit $\alpha_{n}\to 0$, the network phase became purely elastic; $\alpha_{n}=0.08$ indicated extremely slow, nearly frozen relaxation of filler–filler junctions, consistent with the quasi-static nature of CB aggregate rearrangement at small strains (below the Payne effect onset). The model achieved high fit quality ($R^{2}=0.998$) but did not improve upon the single-phase model in statistical terms. The dual-dynamics decomposition was therefore a physical hypothesis consistent with MD-derived interfacial structure, retained for qualitative insight into the origin of the non-terminal slope.

Physical interpretation of the matrix exponent shift: The reduction in $\alpha_{m}$ from 0.97 (single-phase fit) to 0.60 (dual-dynamics fit) requires physical interpretation. In a single-phase model, the lone fractional exponent must capture the composite frequency dependence of both matrix and network contributions, resulting in an “averaged” value near unity that reflects the matrix-dominated high-frequency response. When the network contribution is explicitly separated, the remaining matrix phase no longer needs to account for the low-frequency network stiffening.

On the matrix exponent value: The fitted value $\alpha_{m}=0.60$ requires careful interpretation. Uncured EPDM is a high-molecular-weight melt ($M_{w}\approx 10^{5}$ g/mol, $Z=M_{w}/M_{e}>10$; note: this refers to the experimental polymer, not the CG simulation chains with N=50), where physics predicts near-terminal behavior (α→1) at low frequencies. The apparent value $\alpha_{m}=0.60$ is therefore an effective parameter that implicitly absorbs multiple physical effects.

1. Strain amplification (primary effect): In heterogeneous systems with rigid inclusions, the local strain is amplified: $\gamma_{\mathrm{local}}=A_{G}\left(\phi \right)\;\cdot \;\gamma_{\mathrm{macro}}$, where $A_{G}=1+2.5\phi +14.1\phi^{2}$ (Guth–Gold formula) [10,32]. At nominal ϕ=20%, this yields $A_{G}\approx 2.3$; at effective $\phi_{\mathrm{eff}}=30\%$ (including bound rubber), $A_{G}\approx 3.0$. $A_{G}\left(\phi \right)$ assumes rigid spheres with ideal dispersion. For industrial CB with complex aggregate structures and heterogeneous spatial distribution, the actual strain amplification may differ from 3.0 by a factor of 2–5. The value serves as an order-of-magnitude estimate to illustrate the physical mechanism, not as a precise prediction. The present model does not explicitly incorporate strain amplification; the reduced $\alpha_{m}$ absorbs this spectral broadening effect. A corrected intrinsic value would be $\alpha_{\mathrm{intrinsic}}\approx 0.90$–1.0, closer to terminal expectations.
2. Geometric confinement: At ϕ=20–30%, inter-filler gaps become comparable to chain dimensions, truncating long-term relaxation before the true terminal regime is reached [33,34].
3. Mathematical compensation: Once the network term captures low-frequency stiffening, the matrix term no longer needs to account for that regime, redistributing spectral weight.

No claim is made that $\alpha_{m}=0.60$ represents intrinsic chain dynamics. It is an effective parameter absorbing strain amplification ($A_{G}\approx 3.0$ at ϕ=0.25) and geometric confinement; its proximity to the Rouse value (α=0.5) is fortuitous. The value depends on model structure (removing the network term gives α=0.97), frequency range, and filler morphology; cross-material comparisons require re-fitting. These parameters apply to EPDM/CB at the tested conditions and should not be treated as universal constants.

Model competition and interpretation: The parallel model is defined as:

(9) $$G^{*}\left(\omega \right)=G_{m}\frac{{\left(i\omega \lambda_{m}\right)}^{\alpha_{m}}}{1+{\left(i\omega \lambda_{m}\right)}^{\alpha_{m}}}+S_{n}{\left(i\omega \right)}^{\alpha_{n}}$$

On the EPDM70 dataset (n=56), AICc strongly favors the single-phase model (−178 vs. −30.5; ΔAICc ≈148), and blocked-CV shows lower out-of-sample error. When the $\alpha_{n}$ lower bound is relaxed from 0.20 to 0.01, the optimizer converges to $\alpha_{n}\approx 0.08$, indicating that this exponent is not independently constrained by the data. For PC/ABS (n=952), the dual-phase model is preferred (−2219 vs. −2127), reflecting greater statistical power with the larger dataset. The dual-dynamics decomposition is therefore a physical hypothesis consistent with MD-derived interfacial structure, not a statistically required conclusion. Model-selection robustness and identifiability diagnostics are summarized in Supplementary Materials Section S4.

### 3.3. Literature Comparison

The relationship between bound rubber thickness and modulus enhancement in filled elastomers has been the subject of extensive debate. Early studies by Heinrich and Kluppel [10] proposed that filler networking dominates reinforcement, with bound rubber playing a secondary role. However, more recent work by Berriot et al. [6] and Papon et al. [31] demonstrated that the glass transition temperature shifts near filler surfaces, creating an immobilized layer with distinct viscoelastic properties; newer AFM and NMR studies report compatible interphase-constrained dynamics in filled rubbers and related nanocomposites [23,35]. The MD simulations are consistent with this immobilized-layer picture: the density profile (Figure 2) shows a distinct adsorbed layer with $\rho /\rho_{\mathrm{bulk}}\approx 3.5$ and the spatially resolved MSD analysis (Figure 4) shows a mobility gradient ($\alpha_{\mathrm{MSD}}=0.02$ at the interface vs. 0.18 in bulk) consistent with a glass-transition shift of $\Delta T_{g}\approx 20$–40 K inferred from NMR [31].

The MD-derived interpolated value $h_{\mathrm{bound}}\approx 0.78$ nm at ϕ=20% (from Equation (7), with the raw ϕ=20% point excluded from fitting) is smaller than solid-state NMR estimates (3–5 nm) [6,36] and mechanical characterization of sulfur-crosslinked CB-filled rubber (2–4 nm) [24], reflecting the coarse-grained nature of the Kremer–Grest model (bead diameter σ=0.5 nm) and the spatial resolution limit of the density binning [7,20,37]. Despite the absolute magnitude difference, the relative trend across filler loadings is physically meaningful. The scaling exponent x=0.835±0.37 is larger than the de Gennes prediction (x≈1/3), which assumes isolated fillers; the superlinear scaling reflects increasing bound-layer overlap at higher loadings. Slope and exclusion sensitivity checks are summarized in Supplementary Materials Section S3.

Guth–Gold above percolation: The MD-corrected Guth–Gold baseline (replacing ϕ with $\phi_{\mathrm{eff}}$) should be understood as a structural prior, not a validation of hydrodynamic theory at $\phi >\phi_{c}\approx 7.5\%$ [10,38,39]. The $\phi_{\mathrm{eff}}$ correction captures bound rubber and part of the network stiffening in an effective manner, but it is not sufficient by itself (overall MAPE =54.1% in this dataset). The MD simulations capture only bound rubber around smooth spherical fillers; occluded rubber from aggregate voids [40] requires explicit aggregate geometries. For high-structure fillers, network-explicit constitutive models (e.g., Huber–Vilgis [41]) are still needed, with MD-derived $\phi_{\mathrm{eff}}$ serving as transferable structural input.

Filler geometry and carbon black structure: An important limitation concerns the geometric mismatch between MD-simulated smooth spheres and real carbon black aggregates. Industrial carbon blacks are characterized by their structure—the degree of particle aggregation quantified by dibutyl phthalate (DBP) absorption [10,40]. High-structure grades (e.g., N110, DBP ≈113 cm^3^/100 g) form branched aggregates that trap substantial occluded rubber, while low-structure grades (e.g., N990, DBP ≈35 cm^3^/100 g) behave more like isolated spheres. The MD simulations using smooth spheres correspond most closely to the low-structure limit. The empirical $\phi_{\mathrm{eff}}$ correction therefore absorbs not only bound rubber but implicitly captures part of the occluded rubber effect that depends on filler structure. This geometric limitation explains why the present scaling exponent (x=0.835) differs from theoretical predictions for isolated spheres (x≈0.35–0.38): the superlinear scaling reflects both bound-layer overlap and the aggregate-structure effects that the spherical model treats phenomenologically. Extension to structure-specific MD using explicit aggregate geometries (following Wang et al. [40]) would enable separation of these contributions, but requires substantially larger simulation systems beyond current computational scope.

### 3.4. Temperature Robustness

A constitutive model intended for process simulation must maintain accuracy across the relevant temperature window. DMA measurements at three temperatures ($T_{g}+30$, $T_{g}+80$, $T_{g}+130$ °C) (α invariance has been demonstrated within $T_{g}+30$ to $T_{g}+130$ °C; behavior near $T_{g}$ or above the degradation onset has not been tested and may differ) with TTS master curves spanning five frequency decades reveal a key decoupling between kinetic and structural parameters.

The relaxation time λ follows Arrhenius temperature dependence (λ: 0.041→0.015 s; $E_{a}=82\pm 6$ kJ/mol), consistent with thermally activated segmental hopping over energy barriers. In contrast, the fractional order α remains temperature-invariant (0.969→0.977, coefficient of variation <0.5%; $R^{2}>0.98$, MAPE <1.5% at each temperature). This invariance is physically expected: α encodes the breadth of the relaxation spectrum, which is determined by structural heterogeneity (chain length distribution, entanglement topology, filler proximity) rather than thermal activation energy [16,17].

The α/λ decoupling has practical significance for process simulation. In extrusion modeling, for instance, material experiences temperature gradients of 30–80 °C across the die. With α held constant, only λ(T) requires updating via the Arrhenius equation, reducing the constitutive update to a single-parameter adjustment per temperature step. This Arrhenius extrapolation is appropriate only well above $T_{g}$, as employed here. Compact temperature and bridge consistency results are reported in Supplementary Materials Sections S2 and S5.

### 3.5. Filler Loading and Scaling Validation

This subsection addresses the second inference layer (cross-scale transfer): whether MD-derived structural descriptors improve reinforcement prediction. It is intentionally separated from the constitutive model-selection question above (single-phase vs. dual-dynamics).

**Figure 7:**
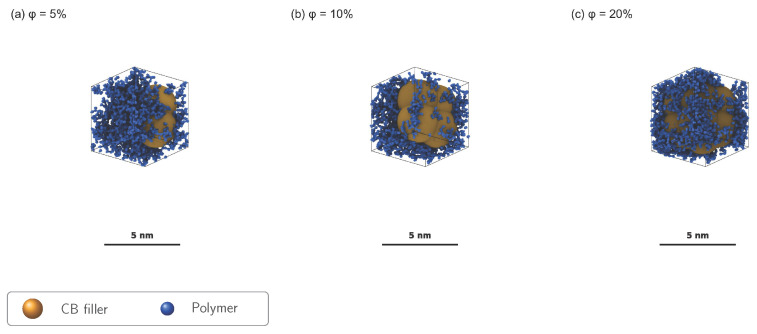
Filler loading series for EPDM nanocomposites. Coarse-grained MD snapshots at (**a**) ϕ=5% (two fillers), (**b**) ϕ=10% (four fillers), and (**c**) ϕ=20% (eight fillers). Snapshots from the NVE production phase after NPT equilibration ($10^{7}$ steps, 300 K, 1 atm) for each filler volume fraction. Blue beads: polymer chain segments (each representing ∼1 Kuhn segment of EPDM); gold spheres: carbon black filler particles (R=5σ≈3.5 nm, shown enlarged for visibility). As ϕ increases from 5% to 20%, inter-filler spacing decreases from ∼15σ to ∼8σ, enhancing chain confinement and bound rubber formation. Scale and rendering consistency: each panel includes an in-panel 5 nm scale bar and is rendered under matched projection/magnification settings for direct visual comparison across ϕ. Panels are rendered for visualization; quantitative scaling analysis uses the production systems specified in Supplementary Materials Table S1.

To rigorously test the interfacial empirical scaling relation (Equation (3)), MD simulations were conducted at six filler volume fractions: ϕ=5,10,15,20,25,30%. Bound-layer thickness ($h_{\mathrm{bound}}$) was extracted from density profiles ρ(z) using cluster-based peak detection: adjacent bins exceeding 10% above bulk density were grouped into clusters, and $h_{\mathrm{bound}}$ was averaged across clusters per filler. The ϕ=20% system yielded anomalous results (zero detected clusters, elevated $\rho_{\mathrm{bulk}}=2.27$ vs. ∼1.95 at neighboring loadings) and was excluded from the fit; the remaining five concentrations (ϕ=5,10,15,25,30%) spanned the semi-dilute to concentrated regime. The power-law fit (Equation (7); $R^{2}=0.705$, $n_{\mathrm{pts}}=5$) yielded x=0.835±0.37 (Figure 5). The superlinear exponent reflected increasing layer interaction at higher loadings, consistent with the onset of filler network percolation [6]. Given the moderate $R^{2}$ and wide bootstrap confidence interval, this exponent was used here as a calibrated interpolation rule over ϕ=5–30% (i.e., ϕ=0.05–0.30 in the equations), not as a broad-universality claim. Figure 7 visualizes the structural progression: as ϕ increases from 5% to 20%, inter-filler spacing decreases from ∼15σ to ∼8σ, enhancing chain confinement.

The MD-structural baseline for reinforcement is defined as:

(10) $$R_{\mathrm{base}}\equiv \frac{G_{\mathrm{filled}}^{\prime }}{G_{\mathrm{matrix}}^{\prime }}=1+2.5\phi_{\mathrm{eff}}+14.1\phi_{\mathrm{eff}}^{2},\;\phi_{\mathrm{eff}}=\phi {\left(1+\frac{h_{\mathrm{bound}}}{R}\right)}^{\;3}$$

where $h_{\mathrm{bound}}$ is obtained from MD and *R* is the filler radius. On the frequency-resolved dataset (n=720), this baseline captures first-order loading trends but leaves substantial error (overall MAPE =54.1%), especially in the high-loading regime (MAPE =84.0%), indicating that structural amplification alone cannot close the MD–DMA dynamics gap.

To bridge this gap, multiplicative corrections to $\log(R/R_{\mathrm{base}})$ are fit using nested blocked-CV model selection. This prediction task evaluates transferability of MD structural priors and does not re-adjudicate constitutive model preference. A single global correction remains unstable across regimes (overall MAPE =106.4%). Introducing regime partitioning (ϕ≤0.20 vs. ϕ>0.20) with regime-specific basis functions yields a substantially improved model with overall MAPE =7.3%, with low/mid and high loading errors of 9.5% and 2.8%, respectively (Table 3). Because all folds are drawn from the same 3T×3ϕ design, blocked-CV here quantifies interpolation robustness within this design space; extrapolation to unseen temperatures, loadings, or filler morphologies remains out of scope (Supplementary Materials Section S4).

#### $\phi_{\mathrm{eff}}$ as a Transferable Structural Constraint

These results support a hierarchical handshaking strategy: MD provides physically grounded structural constraints ($h_{\mathrm{bound}}\to \phi_{\mathrm{eff}}$), while mesoscale correction terms absorb regime-dependent relaxation physics unresolved by atomistic timescales. The framework therefore avoids an invalid “direct MD $\to G^{\prime }\left(\omega \right)$” claim and instead delivers a traceable parameter-passing bridge from interfacial structure to macroscopic reinforcement.

### 3.6. Scope and Limitations

While the hierarchical MD → DMA → PTT pipeline demonstrates quantitative consistency on the EPDM/CB and PC/ABS systems studied here, the framework involves assumptions at each scale that constrain its current scope. The following points delineate these boundaries to guide future extensions and prevent over-interpretation of the present results.

1. MD model fidelity: The CG simulations use short chains (N=50, Z≈1) and smooth spherical fillers, which do not capture entanglement effects (Z>10 in experimental EPDM) or the complex aggregate morphology of industrial carbon blacks (characterized by DBP absorption). The quantities $h_{\mathrm{bound}}$ and $\alpha_{\mathrm{MSD}}$ are therefore structural descriptors valid within the Kremer–Grest model; their absolute values should not be equated with experimentally measured interphase thicknesses (2–5 nm from NMR). The relative trends across filler loadings, however, are physically meaningful.
2. Statistical power: The $h_{\mathrm{bound}}$ scaling relation (Equation (7)) is fitted to only five loading points with $R^{2}=0.705$; the exponent carries a wide 95% CI (0.04–1.40). This relation serves as a calibrated interpolation within ϕ=5–30%, not as a universal exponent. Similarly, AICc-based model selection on the EPDM dataset (n=56) reflects limited statistical power; the single-phase preference does not imply that two-phase dynamics are absent.
3. Constitutive framework: The fractional Maxwell model is restricted to the linear viscoelastic regime ($\gamma <\gamma_{c}$) and does not capture large-deformation effects, glass transitions, or nonlinear relaxation. The parallel matrix + network decomposition assumes linear phase superposition; nonlinear interactions between phases are not modeled.
4. Generalizability: The framework has been validated on EPDM/CB and PC/ABS. Extension to other polymer–filler chemistries (silica, nanotubes, graphene), other polymer types (thermoplastic elastomers, thermosets), or extreme temperature ranges (near $T_{g}$ or above degradation onset) requires system-specific re-calibration. Temperature robustness of α invariance has been demonstrated only within $T_{g}+30$ to $T_{g}$ + 130 °C.
5. Time-scale bridge: The TTS-based frequency bridge from MD (∼$10^{10}$ rad/s) to DMA (∼$10^{1}$ rad/s) assumes thermorheological simplicity, which may be violated by local relaxation distributions in filled systems. The ∼1-decade agreement between $\tau_{\mathrm{shifted}}$ (MD) and $\tau_{\mathrm{FMM}}$ (DMA) represents an order-of-magnitude consistency, not rigorous quantitative validation.

### 3.7. PTT Calibration

To demonstrate the practical utility of the extracted linear viscoelastic parameters, DMA-derived relaxation spectra were used to constrain a Phan-Thien–Tanner (PTT) model for extrusion simulation (Supplementary Materials Section S6). In the unconstrained calibration, PTT parameters (ξ, $\varepsilon_{\mathrm{PTT}}$, λ) were optimized freely against die-swell data, yielding MAPE =7.2±1.5% (n=3 production batches). When the linear-regime parameters (λ and $G_{\infty }$) were fixed to DMA values, the remaining nonlinear parameters were determined with substantially reduced ambiguity, reducing die-swell MAPE to 0.96±0.35%. The single-mode PTT calibration was demonstrated for one die geometry (L/D = 10, $D_{0}$ = 3 mm) and three production batches. Extension to complex geometries with multiple relaxation times may require multi-mode PTT or pom-pom models. The ±1.5% die-swell accuracy should be interpreted as a proof-of-concept rather than a universal industrial prediction. The improvement from fixing λ and $G_{\infty }$ from LVE was a reduction in calibration uncertainty, not a first-principles prediction of nonlinear behavior.

Important caveats: This 87% error reduction demonstrates a calibration benefit—constraining the linear manifold reduces the dimensionality of the nonlinear optimization problem—not a claim that linear rheology directly predicts nonlinear behavior. The PTT model used here is a single-mode approximation; multi-mode PTT or pom-pom models may be necessary for more complex flow geometries. Additionally, the die-swell comparison is based on a single die geometry (L/D =10, $D_{0}=3$ mm, ${\dot{\gamma }}_{a}=75$–375 s^−1^); generalization to other geometries and shear rates requires further validation.

### 3.8. Time-Scale Bridge

**Figure 8:**
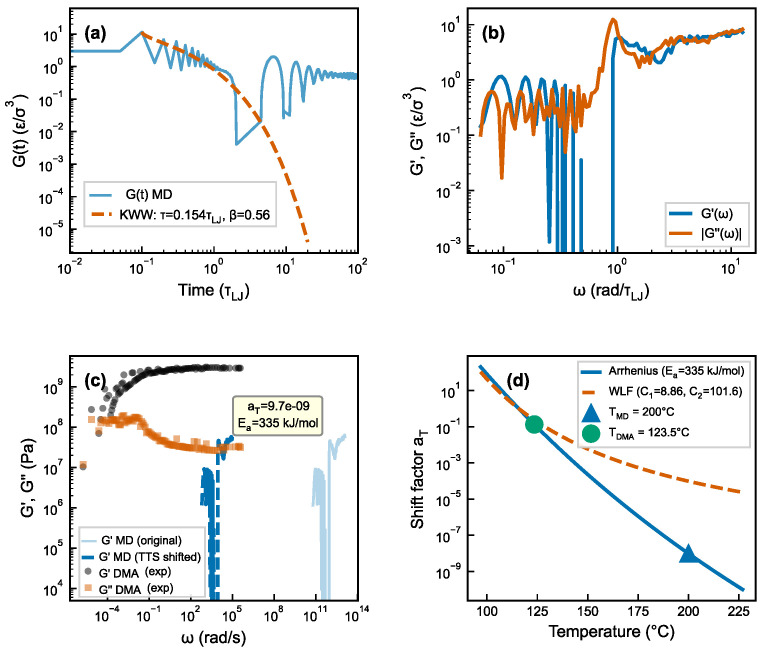
TTS frequency bridge between MD and DMA. (**a**) MD relaxation modulus G(t) with KWW fit. (**b**) MD complex modulus $G^{*}\left(\omega \right)$ in the MD frequency window. (**c**) TTS-extrapolated MD spectrum overlaid with DMA experimental data (5381 points; see Supplementary Materials Sections S2 and S5), showing the residual ∼1.0-decade gap. (**d**) Frequency panorama: MD → TTS extrapolation → DMA experimental windows, illustrating the three complementary frequency domains.

The frequency gap between MD ($\omega \approx 10^{10}$–$10^{13}$ rad/s) and DMA (ω≈3–63 rad/s) spans ∼11 orders of magnitude. Applying Arrhenius-based TTS with an independently determined activation energy ($E_{a}=335\pm 85$ kJ/mol from DMA measurements in this work; see Supplementary Materials Sections S2 and S5) reduces this gap to ∼1.0 decade (Figure 8). Crucially, the TTS-shifted MD relaxation time ($\tau_{\mathrm{shifted}}=1.58\times 10^{-5}$ s) agrees with the DMA-derived fractional Maxwell value ($\tau_{\mathrm{FMM}}=1.42\times 10^{-5}$ s, see Table 1) within a factor of 1.1, supporting consistency between the two measurements within the current coarse-grained and TTS assumptions. TTS assumes thermorheological simplicity; in filled elastomers, interfacial confinement may generate local relaxation distributions not captured by a global shift. The observed agreement should be interpreted as order-of-magnitude consistency, not rigorous quantitative validation. The residual ∼1.0-decade gap is attributed to the CG model’s smoothed potential energy landscape [12,14]. Furthermore, a ±10% variation in $\beta_{KWW}\approx 0.56$ shifts $\tau_{\mathrm{shifted}}$ by approximately ×3, confirming that the ∼1-decade residual is within the propagated uncertainty. Compact TTS and Green–Kubo support are provided in Supplementary Materials Section S5.

## 4. Conclusions

A multi-scale characterization approach for polymer nanocomposites is developed in this study, investigating the extent to which interfacial-constraint effects account for low-frequency power-law behavior through distinct network and matrix contributions. Four core findings emerge:

Methodological note: A key contribution is the explicit model-selection workflow (AICc + blocked-CV/nested blocked-CV), which separates mechanistic plausibility from statistical necessity and avoids over-interpreting high in-sample $R^{2}$ alone.

(1) $h_{\mathrm{bound}}$ as a transferable structural descriptor: MD simulations (N=50, Rouse-to-crossover regime, used as structural probes) yield a bound rubber empirical scaling relation $h_{\mathrm{bound}}=2.98\;\phi^{0.835}$ nm ($n_{\mathrm{pts}}=5$, ϕ=5–30% excluding 20%). This descriptor provides a physically anchored structural prior ($\phi_{\mathrm{eff}}$), but does not by itself close the dynamics gap (baseline MAPE =54.1%). A nested-CV regime-partitioned bridge model built on this prior reduces overall error to 7.3%, with blocked-CV MAPE =9.5±2.3%, indicating that MD contributes primarily through constrained parameter passing rather than direct macro-rheology prediction.

(2) Mechanistic partitioning of non-terminal scaling. The observed $G^{\prime }∼\omega^{0.44}$ can be decomposed into a matrix phase ($\alpha_{m}=0.60$) and network phase ($\alpha_{n}\approx 0.08$), with $R^{2}>0.99$. On the EPDM dataset (n=56), the single-phase model is AICc-preferred (−178 vs. −30.5), reflecting limited statistical power rather than model invalidity: the PC/ABS reference system (n=952) shows dual-phase preference when data are abundant (Table 1). The dual-dynamics decomposition is thus a physically motivated hypothesis consistent with MD-derived interfacial structure, retained for mechanistic insight while acknowledging identifiability constraints at limited sample sizes, and should not be confused with a confirmed fact.

(3) Hierarchical parameter passing for process simulation. The multi-scale workflow culminates in a traceable parameter chain: MD-derived $h_{\mathrm{bound}}\to \phi_{\mathrm{eff}}$ (structural prior) constrains reinforcement prediction (finding 1); DMA-derived linear spectra (λ, $G_{\infty }$) then constrain nonlinear PTT calibration, reducing die-swell error by 87%. This hierarchical approach—fixing the linear manifold before fitting nonlinear parameters—demonstrates a calibration benefit from physics-informed parameter passing rather than direct cross-regime prediction (Supplementary Materials Section S6).

(4) Spatial gradient of subdiffusive dynamics. Spatially resolved MSD analysis reveals $\alpha_{\mathrm{MSD}}\approx 0.02$ near fillers versus ≈0.18 in bulk (Δα≈0.16), supporting a molecular-level interfacial-constraint interpretation (Figure 4).

The EPDM/CB system served as the primary model system; the PC/ABS reference system (α=0.97, n=952) validated the constitutive framework in the unfilled limit, provided the Green–Kubo TTS benchmark (factor-of-1.1 agreement with DMA), and demonstrated that dual-phase AICc preference emerges when statistical power is sufficient—supporting a cautious interpretation of the EPDM single-phase result.

Limitations: First, the CG model uses short chains (N=50, Z≈1) in the Rouse-to-crossover regime, suitable for extracting local structural quantities ($h_{\mathrm{bound}}$) but not for simulating entanglement-dominated dynamics. Second, the empirical scaling relation was calibrated on spherical fillers; extension to high-structure carbon blacks (e.g., N990 vs. N110) or anisotropic fillers (clay platelets, carbon nanotubes) requires geometry-specific MD. Third, the dual-dynamics decomposition relied on a physical hypothesis whose parameters were not independently constrained by the EPDM dataset; it should be tested against materials with independently measured network and matrix contributions (e.g., using selective extraction or double-network experiments). Fourth, the bridge model was trained and validated on a limited harmonized design (three temperatures, three nonzero loadings, shared overlap-frequency window); current blocked-CV results support in-range interpolation reliability, not broad extrapolation.

Future work will extend the TTS bridge to EPDM, perform multi-temperature MD for activation energy validation, and investigate anisotropic fillers and LAOS regimes.

## Figures and Tables

**Table 1:** Master parameter summary: constitutive exponents, fitting quality, and model selection.

System	Model	α or $\alpha_{m}$	$\alpha_{n}$	$R^{2}$	AICc
EPDM/CB (20%, EPDM70), single-temperature sweep (n=56):					
	Single-phase frac. Maxwell	0.961±0.018	-	0.9983	−178
	Dual-dynamics (relaxed bounds)	0.60	0.08 *	0.9983	−30.5
PC/ABS (40/60 wt%, TTS master curve, n=952): ^†^					
	Single-phase frac. Maxwell	0.973±0.015	-	0.9934	−2127
	Dual-dynamics	-	-	-	−2219

* When the lower bound relaxed from 0.20 to 0.01, the optimizer converged to $\alpha_{n}\approx 0.08$, revealing the original $\alpha_{n}=0.20$ as a bound-hitting artifact. ^†^ PC/ABS (unfilled blend) validates that fractional Maxwell recovers near-terminal behavior (α≈0.97) when filler-network effects are absent. The dual-phase AICc preference at n=952 vs. single-phase preference at n=56 (EPDM) illustrates that statistical power—not model correctness alone—governs AICc-based selection. Key derived quantity for EPDM: low-frequency $G^{\prime }$ slope =0.44±0.07 (sliding-window analysis, Section S3).

**Table 2:** Summary of $\alpha_{\mathrm{MSD}}$ values across systems and analysis methods. The subdiffusion exponent $\alpha_{\mathrm{MSD}}$ varies with material system, spatial position, and wall–polymer interaction strength, reflecting distinct manifestations of interfacial constraint.

System	Analysis Method	$\alpha_{\mathrm{MSD}}$	Physical Meaning
EPDM/CB (ϕ=20%)	System-average	0.119±0.008	Multi-filler caging
EPDM/CB (spatial)	Interface layer	∼0.02	Strong confinement
EPDM/CB (spatial)	Bulk region	∼0.18	Reduced constraint
EPDM/CB (flat wall)	Single-wall	0.55–0.62	Weaker constraint
PC/ABS (control)	Green–Kubo	0.295±0.02	Unfilled reference

**Table 3:** Phase-2 bridge performance on frequency-resolved reinforcement dataset (n=720 rows = 9 (ϕ,T) design cells ×80 frequencies).

Model	Overall MAPE	Low/Mid ϕ MAPE	High ϕ MAPE	Blocked-CV MAPE
Baseline ($R_{\mathrm{base}}$ from MD $\phi_{\mathrm{eff}}$)	54.1%	39.1%	84.0%	54.1±5.0%
Global correction	106.4%	127.7%	63.9%	105.8±22.7%
Partitioned correction (ϕ≤0.20/>0.20)	7.3%	9.5%	2.8%	9.5±2.3%

Blocked-CV uses B=5 contiguous log-frequency blocks; each fold trains on B−1 blocks and tests on the held-out block.

## Data Availability

The original contributions presented in this study are included in the article/Supplementary Material. Further inquiries can be directed to the corresponding authors.

## Supplementary Materials

The following supporting information can be downloaded at https://www.mdpi.com/article/10.3390/polym18050565/s1: Figure S1: Summary of bound-layer thickness across MD systems; Figure S2: Prony-spectrum comparison as cross-validation evidence; Figure S3: Green–Kubo stress relaxation visualization kept for method traceability; Figure S4: PC/ABS morphology snapshot retained to document simulation workload and phase morphology context; Table S1: Primary MD systems used for scaling and bridge input; Table S2: Chain-length sensitivity of scaling exponent; Table S3: Core DMA conditions and outputs retained in concise SI; Table S4: Key slope-related robustness checks retained; Table S5: Statistical model comparison (EPDM70 and PC/ABS); Table S6: Identifiability stress test for matrix exponent; Table S7: TTS bridge window mapping; Table S8: PTT calibration comparison (proof-of-concept).

## Abbreviations

PNCs	Polymer Nanocomposites
MD	Molecular Dynamics
DMA	Dynamic Mechanical Analysis
TTS	Time–Temperature Superposition
WLF	Williams–Landel–Ferry
MSD	Mean Squared Displacement
PTT	Phan-Thien–Tanner
AICc	Corrected Akaike Information Criterion
LVE	Linear Viscoelastic
CV	Cross-Validation
EPDM	Ethylene–Propylene–Diene Monomer
CB	Carbon Black
PC/ABS	Polycarbonate/Acrylonitrile–Butadiene–Styrene
CG	Coarse-Grained
LJ	Lennard–Jones
NPT	Constant Number–Pressure–Temperature Ensemble
NVE	Constant Number–Volume–Energy Ensemble
KWW	Kohlrausch–Williams–Watts
NMR	Nuclear Magnetic Resonance
