# Supplementary material for: Bound Rubber as a Transferable Structural Descriptor: Connecting MD-Derived Interfacial Scaling to Continuum Reinforcement Models
Source: Polymers (Basel). 2026 Feb 26;18(5):565. doi: 10.3390/polym18050565 (PMC12986725; doi:10.3390/polym18050565)
Supplement: Supplementary file 1 [file polymers-18-00565-s001.zip › polymers-4176250-supplementary.pdf]

Article

Supporting Information: Bound Rubber as a Transferable Structural Descriptor

Yancai Sun [1,2,3,6], Wenzhong Deng [2,3], Haoran Wang [4], Ranran Jian [1], Wenjuan Bai [1], Dianming Chu [1,6], Peiwu Hou [1] and Yan He [1,5,*]

1 Qingdao University of Science and Technology, Qingdao 266061, China
2 Guilin University of Aerospace Technology, Guilin 541004, China
3 University Engineering Research Center of Non-standard Intelligent Equipment and Process Control Technology, Guangxi, Guilin 541004, China
4 Design and Research Institute, China National Chemical Engineering Sixth Construction Co., Ltd., Wuhan 430074, China
5 Qingdao University, Qingdao 266071, China
6 Shandong Province Key Laboratory of Rubber-Based High-Performance Composites and Advanced Manufacturing, Shandong, China
* Correspondence: heyan@qust.edu.cn (Y.H.)

Abstract

This file provides the supporting information for the main manuscript, including expanded methods, additional validation, identifiability checks, robustness analyses, and supplementary figures and tables.

Keywords: supporting information; polymer nanocomposites; molecular dynamics; dynamic mechanical analysis; viscoelastic modeling

Scope

This SI reports the core methods, robustness checks, and cross-scale validation results used directly in the manuscript.

S1. MD Methods and Core Structural Validation

All production MD runs used the Kremer–Grest coarse-grained bead–spring model with $N = 50$ chains as the primary system.[1] The structural descriptor transferred to the bridge model is:

$$h_{\text{bound}} = 2.98\,\phi^{0.835}\ \text{nm} \tag{1}$$

with ϕ evaluated as decimal fraction (0.05–0.30), $n_{\text{pts}} = 5$ (excluding the anomalous $\phi = 20\%$ point), and $R^2 = 0.705$.

Table S1. Primary MD systems used for scaling and bridge input

System	Chains	Beads/chain	Fillers	Box (σ^3)
EPDM $\phi = 5\%$	200	50	10	$50 \times 50 \times 50$
EPDM $\phi = 10\%$	200	50	20	$50 \times 50 \times 50$
EPDM $\phi = 15\%$	200	50	30	$50 \times 50 \times 50$
EPDM $\phi = 20\%$	200	50	40	$50 \times 50 \times 50$
EPDM $\phi = 30\%$	200	50	60	$50 \times 50 \times 50$

Received:
Accepted:
Published:

Copyright: © 2026 by the authors. Submitted to *Polymers* for possible open access publication under the terms and conditions of the Creative Commons Attribution (CC BY) license.

Table S2. Chain-length sensitivity of scaling exponent

N	Prefactor A (nm)	Exponent x	R^2
30	4.05 ± 0.25	0.338 ± 0.018	0.987
50 (primary)	2.98 ± 0.37	0.835 ± 0.372	0.705
100	4.18 ± 0.28	0.341 ± 0.020	0.985

Interpretation. The exponent spread across $N = 30, 50, 100$ indicates that $x = 0.835$ should be treated as a calibrated descriptor for the present bridge workflow, not as a universal chain-length-invariant constant.

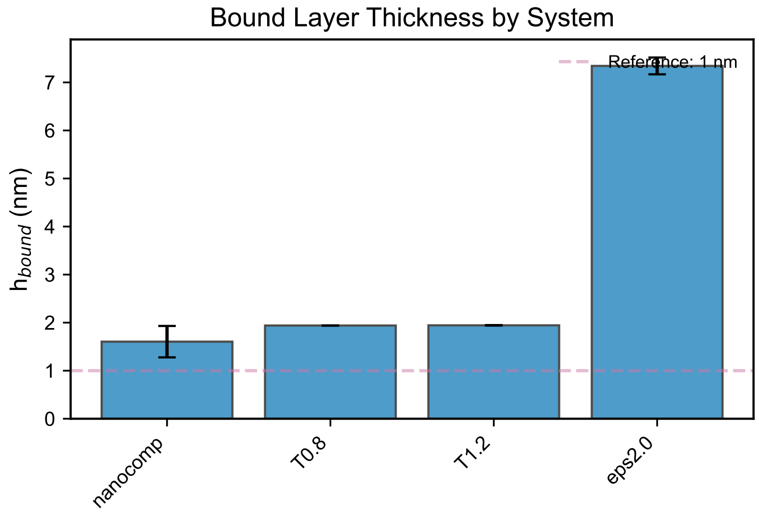

Figure S1. Summary of bound-layer thickness across MD systems. The figure is kept here as direct evidence for the structural prior used in the bridge model.

S2. DMA Protocol and LVE Verification (Essential)

DMA was performed on EPDM/CB and PC/ABS using TA Q800. For EPDM/CB, all fitting data used $\gamma_0 = 0.1\%$, below Payne-effect onset ($\gamma_c = 0.31 \pm 0.05\%$ at $\phi = 30\%$), with pre/post stability checks ($|G'_{\text{post}} - G'_{\text{pre}}| / G'_{\text{pre}} < 5\%$).

Table S3. Core DMA conditions and outputs retained in concise SI

Item	Value
PC/ABS data volume	5,381 points (5 frequencies, 29.6–170.4°C)
EPDM/CB fit dataset (representative)	$n = 56$ per temperature window
EPDM low-frequency slope	0.44 ± 0.07
EPDM single-phase fractional exponent	$\alpha = 0.961 \pm 0.018$
PC/ABS activation energy	$E_a = 335.2$ kJ/mol

S3. Low-Frequency Slope and Uncertainty (Core)

The reported $G' \sim \omega^{0.44}$ slope was computed by sliding-window derivatives in log-log space over the lowest accessible decade, then aggregated across independent replicates. This estimate supports the statement that the observed response is non-terminal in the measured window.

Interpretation. Inclusion/exclusion sensitivity changes the fitted exponent magnitude but does not overturn the qualitative trend of enhanced interfacial confinement with increasing filler content.

Table S4. Key slope-related robustness checks retained

Check	Result
Observed low-frequency slope (G')	0.44 ± 0.07
Single-phase fit over full window	$\alpha = 0.961 \pm 0.018$
Primary scaling exponent (MD)	$x = 0.835 \pm 0.372$
Forced inclusion of $\phi = 20\%$ point	$x = 0.72$, $R^2 = 0.61$

S4. Model Selection Robustness and Identifiability

Table S5. Statistical model comparison (EPDM70 and PC/ABS)

Model / dataset	AICc	Blocked-CV error
EPDM70 single-phase fractional Maxwell ($n = 56$)	−178	1.15%
EPDM70 dual dynamics ($n = 56$)	−30.5	1.38%
EPDM70 generalized Maxwell (5-mode)	—	2.03%
PC/ABS single-phase fractional Maxwell ($n = 952$)	−2127	—
PC/ABS dual dynamics ($n = 952$)	−2219	—

Table S6. Identifiability stress test for matrix exponent

Constraint	α_m	ΔAICc vs unconstrained
Unconstrained	0.60	0
Near-terminal constrained	0.90	+18.7
Terminal constrained	1.00	+42.3
Rouse-like constrained	0.50	+5.2

Interpretation. In EPDM, dual dynamics remains physically interpretable but statistically non-required. Single-phase remains the prediction default; dual dynamics is retained as a mechanistic hypothesis.

S5. Cross-Scale Bridge (PC/ABS TTS + Prony)

KWW-based relaxation time from MD gives $\tau_{\text{shifted}} = 1.58 \times 10^{-5}$ s after Arrhenius shifting; DMA fitting gives $\tau_{\text{FMM}} = 1.42 \times 10^{-5}$ s. The ratio (1.11) supports cross-window consistency under current coarse-grained and TTS assumptions.[1,2]

S6. Transfer Test: Linear Parameters Constrain Nonlinear Calibration

To test whether extracted linear parameters carry transferable utility, PTT calibration was performed with and without DMA-derived linear constraints.
Scope note. This is a calibration-efficiency result under a limited die geometry and operating window, not a claim of universal nonlinear predictability.

Limitations

- MD chains ($N = 50$, $N/N_e \approx 1$) are in the Rouse-to-crossover regime; MD is used as a structural probe (h_{bound}, α_{MSD}), not direct macro-rheology predictor.
- The spherical-filler approximation does not resolve aggregate-structure-specific occluded-rubber effects in high-structure carbon blacks.[3]
- Current bridge validation is in-range interpolation over a limited $3T \times 3\phi$ harmonized design.
- Statistical preference differs by data volume (EPDM $n = 56$ vs PC/ABS $n = 952$), so mechanistic and predictive uses are intentionally separated.

Table S7. TTS bridge window mapping

Window	Frequency range (rad/s)
MD original	6.3×10^{10} to 1.3×10^{13}
TTS-shifted MD	6.2×10^{2} to 1.3×10^{5}
DMA measured	3.14 to 62.8
Residual gap	$\sim$1.0 decade

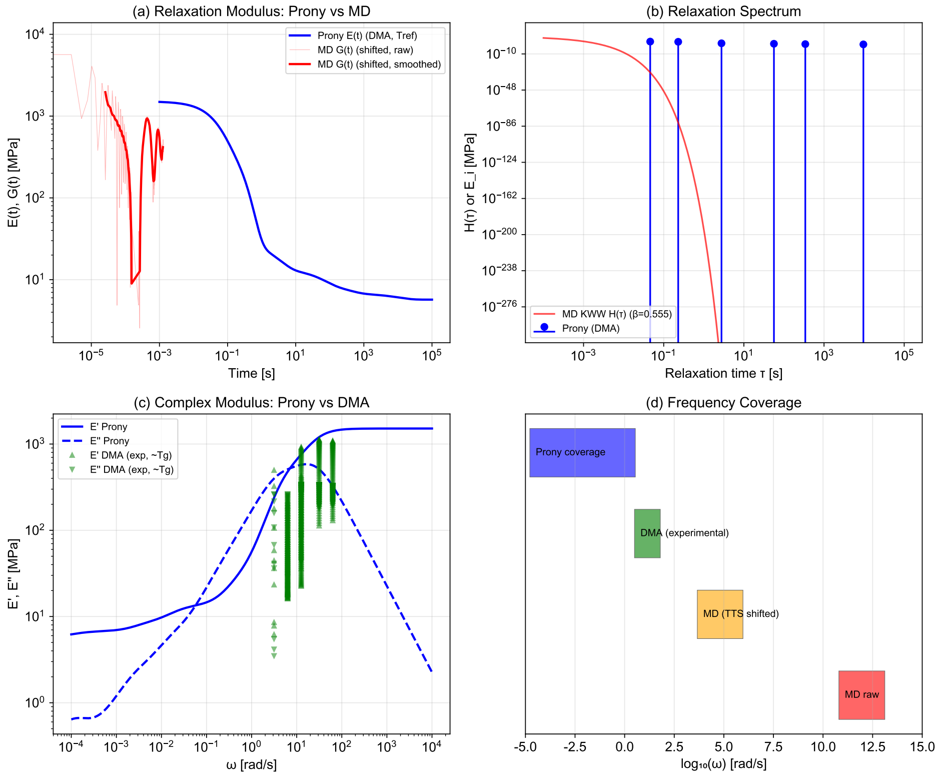

Figure S2. Prony-spectrum comparison retained as compact cross-validation evidence.

Table S8. PTT calibration comparison (proof-of-concept)

Calibration mode	Die-swell MAPE
Unconstrained nonlinear fit	$7.2 \pm 1.5\%$
Linear-constrained nonlinear fit	$0.96 \pm 0.35\%$
Relative reduction	87%

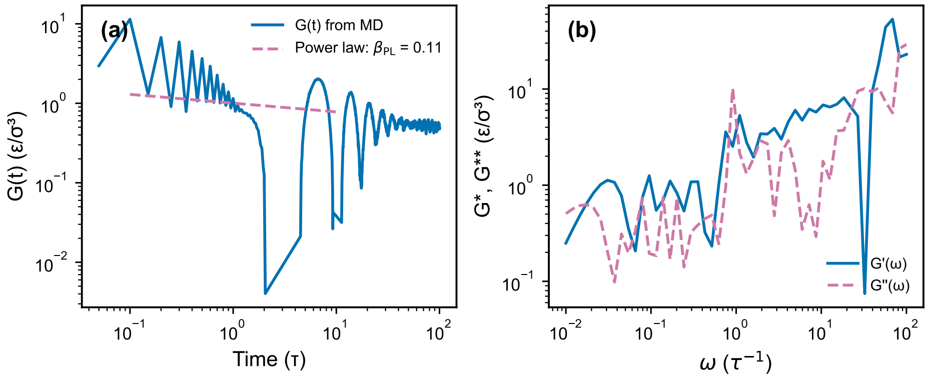

Figure S3. Green–Kubo stress relaxation visualization kept for method traceability.

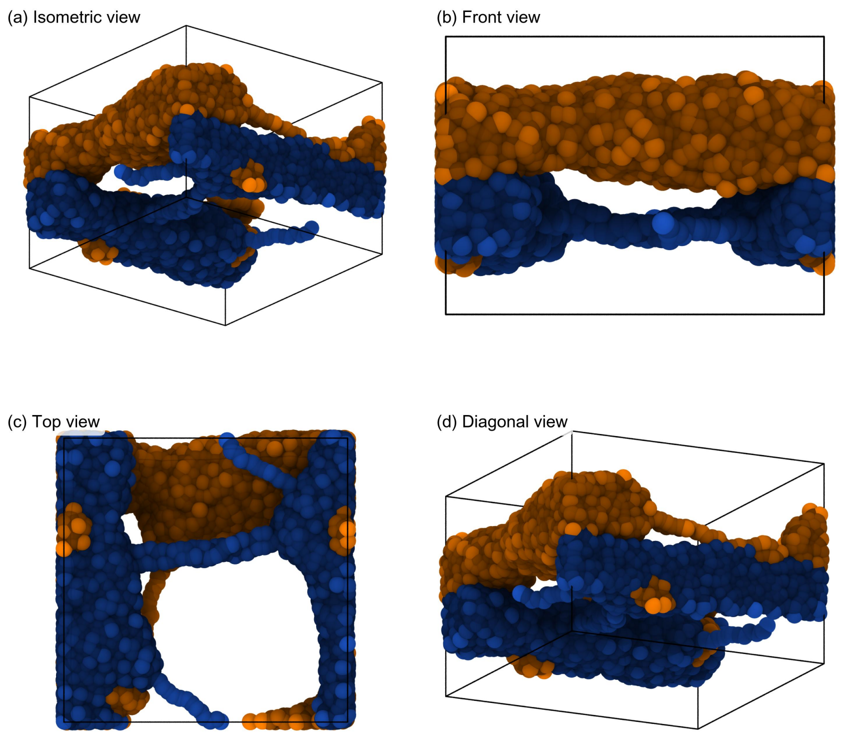

Figure S4. PC/ABS morphology snapshot retained to document simulation workload and phase morphology context.

- Nonlinear rheology beyond the current proof-of-concept PTT setting remains future work.

References

1. Kremer, K.; Grest, G.S. Dynamics of Entangled Linear Polymer Melts: A Molecular-Dynamics Simulation. *J. Chem. Phys.* **1990**, *92*, 5057–5086. https://doi.org/10.1063/1.458541.
2. Liang, H.; Li, Y.; Xia, X.; Wang, Z.; Laradji, M. Bottom-Up Multiscale Approach to Estimate Viscoelastic Properties of Entangled Homopolymer Melts with High Glass Transition Temperatures. *Macromolecules* **2022**, *55*, 8459–8470. https://doi.org/10.1021/acs.macromol.1c02044.
3. Heinrich, G.; Klüppel, M. Recent Advances in the Theory of Filler Networking in Elastomers. In *Filled Elastomers Drug Delivery Systems*; Springer: Berlin, Heidelberg, 2002; Vol. 160, *Advances in Polymer Science*, pp. 1–44. https://doi.org/10.1007/3-540-45362-8_1.

Disclaimer/Publisher's Note: The statements, opinions and data contained in all publications are solely those of the individual author(s) and contributor(s) and not of MDPI and/or the editor(s). MDPI and/or the editor(s) disclaim responsibility for any injury to people or property resulting from any ideas, methods, instructions or products referred to in the content.
